# A simple and short microbiology practical improves undergraduate nursing students’ awareness of bacterial traits and ability to avoid spreading infections

**DOI:** 10.1186/s12909-019-1483-4

**Published:** 2019-02-11

**Authors:** Rika Yano, Torahiko Okubo, Tomoko Shimoda, Junji Matsuo, Hiroyuki Yamaguchi

**Affiliations:** 1 Department of Fundamental Nursing, Faculty of Health Sciences, Hokkaido University, Kita 12, Nishi 5, Kita-ku, Sapporo, 060-0812 Japan; 2 Department of Medical Laboratory Science, Faculty of Health Sciences, Hokkaido University, Sapporo, Japan

**Keywords:** Microbiology, Unseen bacteria, Nurse, Medical education, Practical class, Awareness, Undergraduates

## Abstract

**Background:**

Nurses are responsible for implementing appropriate measures to reduce hospital infections, especially with multidrug resistant bacteria, so nursing students should learn about microbiology. This helps them to understand bacterial dissemination and infectious disease control. Because of tight schedules, however, its teaching is limited in undergraduate nursing classes in Japan. We therefore tested whether a simple short practical session in a microbiology class could help to improve undergraduate nursing students’ awareness of bacterial traits and how to prevent infections.

**Methods:**

This study involved second-grade nursing students (*n* = 76). Two short practical sessions (a total of 3 h, across 2 days) were used to assess the effectiveness of washing or disinfection on hand bacteria in a 16-class microbiology course (total class time was 24 h, plus an exam). Hand bacteria were sampled on LB agar plates with orientation during the first half-day, and the plates examined for colonies with distinct color or morphological traits, and discussed, in the second session, a week later. Questionnaires before and after the exercise were used to assess changes in awareness of unseen bacteria inhabiting around us connecting bacterial traits and how to prevent infections.

**Results:**

The results showed that the practical increased the nursing students’ awareness of fomites (utensils) (*p* = 0.0115), fomites (contact-based) (*p* = 0.0016), habitats (body surface) (*p* = 0.0127), action facilitating hospital infection (*p* = 0.0166), and changes in physical condition caused by bacterial infections (*p* = 0.0136). There were no changes in word associations (*p* = 0.627) or habitats (inside body) (*p* = 0.308). Difficulty score, which is an element in questionnaire psychometric properties, tended to be close to the expected score through the practical, but not statistical significant. In addition, regardless of before or after practical, Cronbach α score, which is an indicator of the reliability among items of multi-choice questions, showed > 0.8, indicating validity of evaluation items. Thus, the student’s awareness of unseen bacteria inhabiting around us was significantly increased as compared to those before practical in microbiology class.

**Conclusions:**

The simple short practical effectively improved nursing students’ awareness of unseen bacteria inhabiting around us in microbiology course, useful for even tight teaching schedules.

## Background

Precautions taken during contact between patients and medical staff and the cleanliness of high-contact surfaces in hospitals are critically important in controlling hospital-acquired infections (HAIs) [1]. Bacterial contamination of surfaces in hospitals surfaces occurs through contact with a range of individuals including visitors, patients, and staff, despite extensive measures to promote cleanliness, including determination of aerobic colony-forming units or detection of ATP on surfaces [2–10]. Nurses are principally responsible for implementing appropriate measures to reduce hospital infections, especially with multidrug resistant bacteria [11]. Improving awareness of unseen bacteria ubiquitously inhabiting around us is directly linked to controlling HAIs. Meanwhile, the contents of microbiology courses in nursing classes are generally based on knowledge because the course lacks in practical work such as forming bacterial culture or colony observation. It is therefore reasonable for nursing students to learn about its practice, at an early stage in their training, undoubtedly helping them to recognize unseen bacteria potentially transmitting everywhere via medical staff or fomites. However there is no consensus about the best way to teach management of standard precautions and precautions based on disease transmission mechanisms to nursing students or other health care students [12].

Although the homogeneous and systematic education system promises to provide high quality classes for those students [13], there is, however, a concern about the poor integration of practice with critical thinking, because of busy class schedules and a focus on theory in lessons [14]. Unfortunately, microbiology course is no exception to this, and inadequate awareness of bacterial traits and how to protect against infection, in particular lacking practical work, is a serious issue. It is therefore considered important to provide effective practical training in microbiology course for nursing students at an early stage of their education, to help them to recognize unseen bacteria around us and understand how to avoid spreading infection. No adequate practical demonstrations in microbiology course have been provided for nursing students, especially undergraduates. Furthermore, it is well accepted that a theoretical framework that can foster experiential learning consisting of a cycle with concrete experience, reflective observation, abstract conceptualization and active experimentation, can generate awareness strongly [15].

In this study, we therefore developed a short and simple practical program (a total of 3 h, over 2 days) that is easily embedded in microbiology courses (which cover a total of 24 h class time plus an exam, and a period of 16 days). We used questionnaires to assess whether this practical program could improve undergraduate nursing students’ awareness of bacterial traits and how to avoid spreading infection.

## Methods

### Study sample

The study involved second-grade nursing students (*n* = 76) from the Faculty of Medicine (Health Sciences), at Hokkaido University. All elements were part of the regular microbiology course.

### Study design

**Fig. 1:**
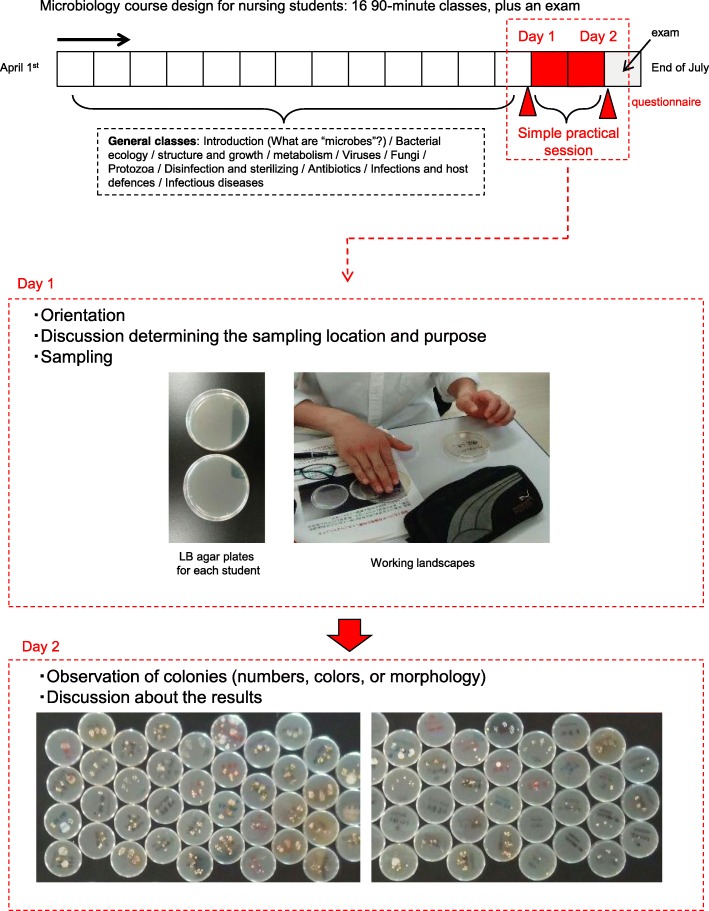
Arrangement of the practical of hand washing with visualizing hand bacteria into microbiology course for nursing students and its evaluation

Figure 1 shows the design of the study. Two short practical sessions (in total, only 3 h, over 2 days, 7 days apart) were used to demonstrate the effectiveness of hand hygiene by washing hand with or without disinfectants (70% alcohol or waterless antiseptic agent) on eliminating hand bacteria. These sessions were part of a basic microbiology course for the nursing students (16 classes totaling 24 h, over the period from April to July, plus an exam). The course covered an introduction, bacterial ecology, structure, growth and metabolism, viruses, fungi, protozoa, disinfection and sterilizing, antibiotics, infections and host defenses, and infectious diseases. The sampling of hand bacteria on an LB (Luria-Bertani) agar plate with orientation took place one day (Fig. 1, “Day 1”), and seven days’ later, the students examined the plates for colonies with distinct color or morphological traits, then discussed their findings (Fig. 1, “Day 2”). For the nursing students, questionnaires (see below) were used to assess changes in awareness of microbiology before and after the practical sessions.

### LB agar plate culture and colony observation

All the agar plates were cultured for 7 days at room temperature. After being carefully sealed with laboratory tape, the cultured plates were carefully observed in the lecture room to assess the color, size, gloss, and number of colonies. Since the sessions took place in a general lecture room, which did not meet specified biosafety levels, the plates could not be opened for observation. It was, however, considered more convenient to carry out the practical in a general lecture room.

### Questionnaires and data expressions

As mentioned above, the questionnaires for the nursing students were administered twice, before and after the practical sessions. The questionnaire included seven general questions (Q1–7), each with eight answer options. The questions covered various aspects of bacteria including word associations, changes in physical condition as a result of infection, fomites (utensil or contact-based material), bacterial habitats (body surface or inside), and actions facilitating hospital infections (see Table 1). Each of the questions allowed multiple answers. For each question, data were expressed as “Improved value Δ%”, calculated as “rate after the practical”−“rate before the practical” . Also, cumulative score of individual students for each of the items in questionnaire were used for estimating psychometric properties (See below).

**Table 1:** Questionnaire items and those choices

Questionnaire items (Q1 to 7): those choices (answers)^a^/ “” ^b^							
Q1. ‘What words images from “bacteria”?’							
dirty	symbiosis	health	harmful	benefical	infection	disease	small
Q2. ‘What physical condition changes images from “bacteria”?’							
pneumonia	vomiting	dermatitis	headache	diarrhea	dizzy	muscle pain	heartbeat
Q3. ‘What fomites (utensils) images from “bacteria”?’							
TV remote controller	PC keyboard	car handle	train hand strap	underground passage	bathtub	kitchen	toilet
Q4. ‘What habitats (body surface) image from “bacteria”?’							
navel	behind the ears	hair	beside the nose	armpit	hips	soles	palm
Q5. ‘What habitats (inside body parts) image from “bacteria”?’							
eyeball	uterus	stomach	brain	lung	intestines	abdominal cavity	pharynx
Q6. ‘What fomites (contact based) image from “bacteria”?’							
stethoscope	toilet seat	public telephone	sumo wrestling	handshake	hand-washing	floor button in elevator	toilet seat with bidet functions
Q7. ‘What actions facilitating hospital infections image from “bacteria”?’							
blood pressure measurements	bedsore treatment	handshake	auscultation	meal	intravenous drip	assistance	blood sampling

^a^For questions that allow multiple answers

^b^For estimating Cronbach’s α score, “correct answer” that we expected are settled up into each of the Questions. Please see the legend of Fig. 3

### Estimation of psychometric properties including score mean, score distribution, Cronbach α score, and difficulty index (DI)

First of all, score mean and its distribution were calculated for estimating Cronbach α score, which is an indicator for estimating the reliability of multi-choice questions based on comparing the score of distribution among questionnaire’s items [16]. The Cronbach α score were estimated by a protocol with an equation [α = (k/k–1)(1–sum of variances of all items/total test variance)] as described previously [16]. In this equation,“k” shows the number of items in questionnaire. Also, to confirm the validity of “difficulty” among items in questionnaire, DI was calculated by a protocol with an equation (number of expected answers/number of all student’s answers) as described previously [16], and then compared among expected answer score, pre-practical score, and after-practical score. All calculations were conducted using Excel for Mac (2015) with Statcel3C.

### Statistical analysis

Comparison between “rate after the practical” and “rate before the practical” was performed using statistical analysis with two-way ANOVA. Comparison among DIs was conducted using a Bonferroni/Dunn test. Additionally, diffract score(score distribution) was estimated using a function with VAR(x) installed into Excel. *P*-value of less than 0.05 was considered significant. Cronbach’s α score of more than 0.8 was considered as having significant validity.

## Results

### The practical’s impact on awareness of unseen bacteria around us among nursing students

**Fig. 2:**
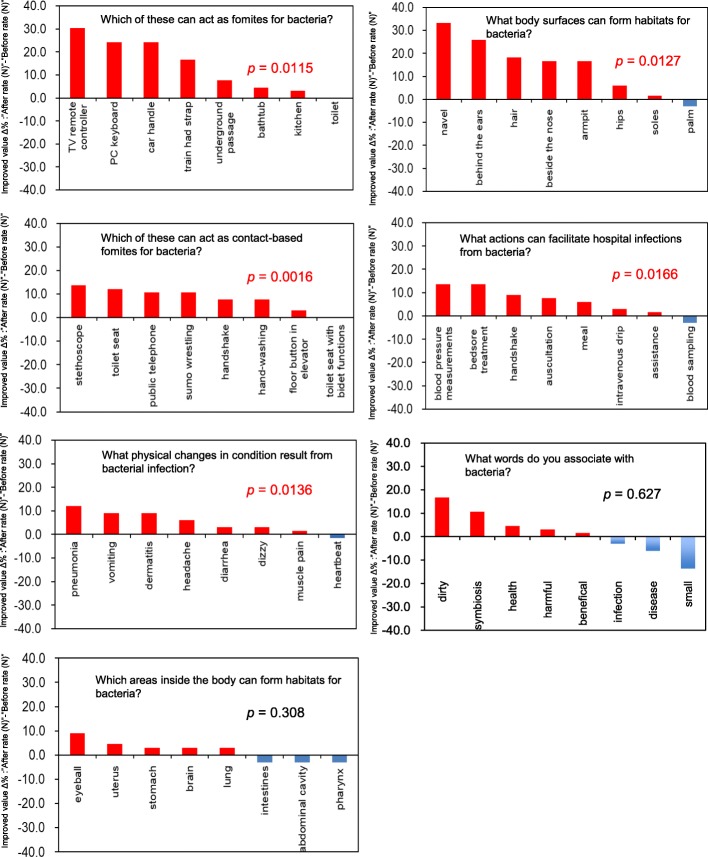
Impact of the practical on awareness of unseen bacteria inhabiting on their hand among nursing students. The x- and y-axes show object names and changes after the practical. For each question, data were expressed as Improved value Δ%, comparing the rate after the practical with the rate before. They were compared using statistical analysis with two-way ANOVA. *P*-value of less than 0.05 was considered significant

To assess whether the practical program had an impact on awareness of unseen bacteria inhabiting around us among the nursing students, we compared their awareness before and after the practical sessions. Figure 2 shows that awareness was significantly increased of fomites (utensils) (*p* = 0.0115), fomites (contact-based) (*p* = 0.0016), habitats (body surface) (*p* = 0.0127), actions facilitating hospital infection (*p* = 0.0166), and physical changes in condition caused by bacterial infections (*p* = 0.0136). There were no changes in word associations (*p* = 0.627) or habitats (inside body) (*p* = 0.308). Thus, the results therefore indicated that even a simple short practical program in microbiology can have a strong impact on increasing awareness about microbiology in particular in terms of unseen bacteria inhabiting around us among nursing students.

### Validity of items in questionnaire on estimating the changes in awareness via practical

**Fig. 3:**
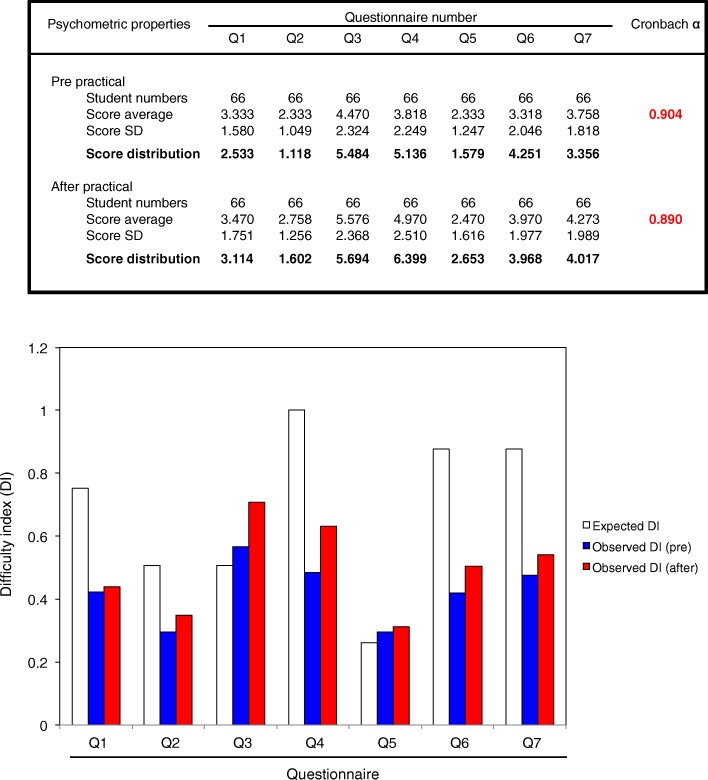
Validity of items in questionnaires on estimating changes in awareness via practical. Upper panel shows psychometric properties including mean score, distribution scores, and Cronbach’s α score. Score distributions show diffract values (See the Methods section). Cronbach’s α score of more than 0.8 was considered as having significant validity. Lower panel shows the comparison of ID (difficulty index) among “Expected DI” (theoretically expected) (White bar), “Observed DI (pre)” (before the practical) (Blue bar), and “Observed DI (after)” (after the practical) (Red bar). The DIs were compared using a Bonferroni/Dunn test, but no significant difference was seen. “Q1–7” shows the items of questionnaires. See Table 1. Additionally, for estimating Cronbach’s α scores, the “correct answers” provided against each of the questions are settled up into each of the Questions as underlined items in Table 1

To verify whether the changes in awareness were a meaningful improvement among nursing students, we estimated psychometric properties including mean, distribution of scores, Cronbach α score and DI. As shown in Fig. 3 (above panel), Cronbach α score on the questionnaires showed > 0.8 (before practical, 0.904; after practical, 0.890), indicating validity of evaluation items in questionnaire. Furthermore, we found that DI tended to be close to the expected score through the practical, but not statistical significant. The result was corresponded to those of awareness in the questionnaires, verifying the items in questionnaire on estimating the changes in awareness via practical.

## Discussion

The undergraduate education system for nurses in Japan, including in microbiology course, lacks integration of practical sessions with critical thinking, because of busy class schedules and a focus on theoretical teaching [13, 14]. Nurses, however, play a key role in preventing hospital-acquired infections, and it is therefore important to provide effective microbiological practical sessions for nursing students at an early stage in their education. We developed a short, simple practical program that is easily embedded in a standard microbiology course. We used questionnaires to assess whether the practical could be useful in improving undergraduate nursing students’ awareness of bacterial traits and how to prevent infections. We found that a simple short practical program in microbiology course can have a strong impact on increasing awareness about unseen bacteria inhabiting around us among nursing students. This suggests that it could be used even in classes with tight schedules, and could therefore be an appropriate measure to help reduce hospital infections.

At this stage in their training, the nursing students had learned only basic chemistry, biology and techniques in nursing care, so had limited knowledge and skills. The practical therefore simply consisted of hand sampling on an LB agar plate, and the observation of colonies formed on the plates. No specified biosafety laboratory room or microscopes were required, which meant that the practical could take place in a general lecture room. The practical took only 3 h across two sessions, so the time consumption was not excessive. It also required very little preparation, because each student only required two agar plates, so the burden on staff was limited, and the expense was low (less than 100 yen per student). It could therefore be embedded into any microbiology course at very little cost. Furthermore, the assessment with Cronbach α scores and DIs verified the reliability of items in questionnaire on estimating the changes in awareness via practical, suggesting that it was a meaningful way to improve awareness of microbiology in particular unseen bacteria inhabiting around us among nursing students.

As expected, comparing the questionnaires showed that the practical improved awareness of unseen bacteria inhabiting around us among nursing students. Awareness was significantly increased of fomites (utensils and contact-based), habitats (body surface), actions facilitating hospital infection, and physical changes in condition caused by bacterial infections, but not in word associations or habitats (inside body). It is well-known that visualizing structures or materials can improve students’ awareness of specific lecture topics, enabling them to better understand lectures [17, 18]. It has, for example, been reported that the oral microbiology lab curriculum appears to improve the quality of oral medicine education and help to cultivate high-quality innovative medical talents [19]. Experiential courses with an emphasis on patient-physician/dentist communication plays a significant role in early on during pre-clinical medical and dental studies [20]. These studies therefore suggest that a combined lecture and practical approach could improve understanding of specific subjects. The observation of colonies may not be surprising, but being able to visualize previously-unseen life through the observations could enable the students to make a connection between those images and more familiar objects such as TV remote controls or stethoscopes. However, word associations and the inside of the body are harder to connect to the observed images, which may explain why the practical had no impact on the awareness of these areas among the nursing students. This may explain the necessity of advanced practical for acquiring deeper knowledge about infectious diseases to nursing students.

## Conclusion

Altogether, we conclude that the simple and short practical in microbiology course offered to nursing students effectively improved their awareness of unseen bacteria inhabiting around us. Meanwhile, this practical has a limitation that this study provided only the second-grade nursing students class, lacking comparison to those of other nursing courses. Further study should be needed to clarify our conclusion.

## Abbreviations

ANOVA: Analysis of variance
ATP: Adenosine triphosphate
DI: Difficulty index
HAIs: Hospital-acquired infections
